# How Probable Cognitive Impairment Shapes Fall Risk and Physical Function After 12 Months

**DOI:** 10.1093/geroni/igaf122.2027

**Published:** 2025-12-31

**Authors:** Laura Himmelmann, Tania Zieschang, Tim Stuckenschneider

**Affiliations:** The Carl von Ossietzky University of Oldenburg’s School of Medicine and Health Sciences, Oldenburg, Niedersachsen, Germany; The Carl von Ossietzky University of Oldenburg’s School of Medicine and Health Sciences, Oldenburg, Niedersachsen, Germany; The Carl von Ossietzky University of Oldenburg’s School of Medicine and Health Sciences, Oldenburg, Niedersachsen, Germany

## Abstract

Older adults with cognitive impairment (OACI) experience more severe fall-related sequelae and have a higher risk of recurrent falls than cognitively healthy older adults (OACH). Despite the high risk of decline in physical function following fall-related emergency department (ED) visits, targeted falls prevention interventions remain limited. Therefore, this study examines the trajectories of physical function and recurrent falls in OACH and OACI. Older adults (≥60 years) were recruited shortly after a severe fall that required an ED visit and were followed up for 12 months. Follow-up included monthly telephone calls and three home visits to assess recurrent falls, functional performance measured by self-preferred, three-meter overground gait speed (m/s), and cognition (MoCA). After twelve months, 116 OACI (mean age 77.1± 8.4; MoCA: 21.8±1.9) experienced more recurrent falls (n = 61/116, p = 0.01) compared to 126 OACH (mean age 70.7±7.8; MoCA: 26.8±1.4; recurrent falls 46/126). ANCOVA indicated a significant difference between the groups with worse performance in OACI (p < 0.001), although both groups showed cognitive improvement over time (p < 0.001). Physical function including gait speed remained stable, with both groups demonstrating persistently slow gait speed at 12 months (OACI: 1.0±0.3; OACH: 1.1±0.3 m/s; p = 0.51). Since physical function remained unchanged, the high fall rate may be more closely linked to deficits in attention or executive function. Nevertheless, a longer follow-up is needed to identify the clinical relevance of these findings. Future studies should explore cognitive training and dual-task exercise interventions to mitigate fall risk in this vulnerable population.

